# Supplementary material for: Influence of Auricularia cornea Polysaccharide Coating on the Stability and Antioxidant Activity of Liposomes Ginsenoside Rh2
Source: Foods. 2023 Oct 29;12(21):3946. doi: 10.3390/foods12213946 (PMC10647797; doi:10.3390/foods12213946)
Supplement: Supplementary file 1 [file foods-12-03946-s001.zip › foods-2631512-supplementary.pdf]

foods

MDPI

Supplementary tables:

Table.S1. Response surface design factors and levels.

Level	Factor			
	A	B	C	D
-1	2:1	1:10	40	30
0	1:1	1:15	50	40
1	1:2	1:20	60	50

Table.S2. Experimental design and response value results.

Test No.	Factor				response
	Film to material ratio	Rh2: Soy lecithin(w:w)	Hydrate temperature(℃)	Hydrate time（min）	EE（%）
1	1:1	1:15	50	40	88.47
2	1:2	1:10	50	40	87.94
3	1:2	1:15	50	30	87.74
4	1:1	1:10	60	40	87.53
5	1:1	1:20	50	40	89.99
6	1:1	1:20	60	30	87.17
7	2:1	1:10	50	40	87.46
8	1:1	1:10	40	30	86.42
9	1:1	1:20	50	40	88.92
10	1:2	1:20	50	50	87.96
11	1:1	1:20	40	30	86.92
12	2:1	1:20	50	30	86.82
13	1:1	1:20	40	30	86.96
14	1:1	1:20	50	40	89.74
15	1:1	1:15	40	40	87.36
16	1:2	1:20	60	40	87.26

17	2:1	1:20	50	50	88.38
18	1:1	1:15	50	30	87.37
19	1:1	1:15	50	50	87.09
20	2:1	1:20	40	40	87.28
21	1:2	1:20	40	40	87.95
22	2:1	1:15	50	40	87.6
23	2:1	1:20	60	40	86.91
24	1:1	1:20	50	40	89.89
25	1:1	1:20	50	40	89.09
26	1:1	1:10	40	40	87.47
27	1:1	1:10	50	50	87.56
28	1:1	1:20	60	50	87.93
29	1:2	1:15	50	40	88.62

Table.S3. Variance analysis of quadratic polynomial model.

Source	Sum of squares	df	Mean square	F value	P value
Model	21.98	14	1.57	8.15	0.0002
A-Film to material ratio	0.7459	1	0.7459	3.87	0.0692
B-Rh2: Soy lecithin	0.1042	1	0.1042	0.541	0.4742
C-Hydrate temperature	0.0064	1	0.0064	0.0331	0.8583
D-Hydrate time	0.6696	1	0.6696	3.48	0.0833
AB	0.0584	1	0.0584	0.3031	0.5906
AC	0.0256	1	0.0256	0.1329	0.7209
AD	0.4489	1	0.4489	2.33	0.1491
BC	0.0124	1	0.0124	0.0646	0.8031
BD	0.0173	1	0.0173	0.09	0.7685
CD	0.0202	1	0.0202	0.1048	0.7509
A2	3.84	1	3.84	19.96	0.0005
B2	5.35	1	5.35	27.76	0.0001
C2	7.35	1	7.35	38.14	< 0.0001

D2	6.46	1	6.46	33.52	< 0.0001
Residual	2.7	14	0.1926		
Lack of Fit	1.74	9	0.1939		
Pure Error	0.9517	5	0.1903		
The sum	24.68	28			
